# Cultural engagement and incident depression in older adults: evidence from the English Longitudinal Study of Ageing

**DOI:** 10.1192/bjp.2018.267

**Published:** 2019-04

**Authors:** Daisy Fancourt, Urszula Tymoszuk

**Affiliations:** 1 Wellcome Research Fellow, Department of Behavioural Science and Health, University College London, UK; 2 Research Associate, Centre for Performance Science, Royal College of Music and Imperial College London; and Research Department of Epidemiology and Public Health, University College London, UK

**Keywords:** Depressive disorders, epidemiology, psychosocial interventions

## Abstract

**Background:**

There is a recognised need for the identification of factors that might be protective against the development of depression in older adults. Over the past decade, there has been growing research demonstrating the effects of cultural engagement (which combines a number of protective factors including social interaction, cognitive stimulation and gentle physical activity) on the treatment of depression, but as yet not on its prevention.

**Aims:**

To explore whether cultural engagement in older adults is associated with a reduced risk of developing depression over the following decade.

**Method:**

Working with data from 2148 adults in the English Longitudinal Study of Ageing who were free from depression at baseline, we used logistic regression models to explore associations between frequency of cultural engagement (including going to museums, theatre and cinema) and the risk of developing depression over the following 10 years using a combined index of the Centre for Epidemiological Studies Depression Scale (CES-D) and physician-diagnosed depression.

**Results:**

There was a dose–response relationship between frequency of cultural engagement and the risk of developing depression independent of sociodemographic, health-related and social confounders. This equated to a 32% lower risk of developing depression for people who attended every few months (odds ratio (OR) = 0.68, 95% CI 0.47–0.99, *P* = 0.046) and a 48% lower risk for people who attended once a month or more (OR = 0.52, 95% CI 0.34–0.80, *P* = 0.003). Results were robust to sensitivity analyses exploring reverse causality, subclinical depressive symptoms and alternative CES-D thresholds.

**Conclusions:**

Cultural engagement appears to be an independent risk-reducing factor for the development of depression in older age.

**Declaration of interest:**

None.

Mental health is an important determinant of successful ageing and longevity. It is, however, prone to decline with age because of life events and circumstances commonly experienced by older adults such as bereavement, lone living, impoverished social interactions, poor health, retirement and worsening economic condition.^1^ In England, it has been estimated that approximately one in four people aged 65 or over are depressed, with the prevalence of depressive symptoms increasing with age.^2^ Depression in older age is also commonly underdiagnosed and undertreated^3^ and is associated with a higher risk of dementia, diabetes, cardiovascular disease, stroke and both specific and all-cause mortality.^4^^–^^8^ In light of this, there has been much research undertaken to identify factors that can protect against the development of depression, including social networks and social support,^9^ physical activity^10^ and cognitive stimulation.^11^ However, despite this, there is a recognised lack of effective multimodal psychosocial interventions for the prevention of depression in older adults.^12^

## Cultural engagement and mental health

Over the past decade, there has been growing research demonstrating the effects of cultural engagement on depression. This has included studies of active cultural engagement (such as singing, dancing or doing artistic activities)^13^^–^^15^ and receptive cultural engagement (such as visiting museums and galleries).^16^^,^^17^ However, to date, much of this research has centred on the impact of cultural engagement on recovery for people with depression or on depressive symptomatology in the general population. There remains little research into whether cultural engagement can itself act as a risk-reducing factor for the development of depression, and therefore play a preventative role. However, cultural engagement combines many risk-reducing factors for depression incidence, which suggest that it could be a protective factor. For example, cultural engagement includes social interaction (either through visiting with friends or interaction with other attendees or staff), which can be a source of social support and act as buffer stress, and thereby reduce the onset and progression of depressive symptoms.^18^ Going to cultural venues is also a way of reducing sedentary behaviour, which is associated with depression, partly through increased inflammatory responses.^19^ Furthermore, the emotional response to cultural activities such as music has been found to involve brain regions critical to the processing of positive emotions and reward.^20^ Cultural activities also require cognitive and perceptual engagement, which itself is associated with lower levels of depression.^21^ Besides, cultural engagement has been found to support coping behaviours in the face of physical health challenges.^22^

However, despite these promising theoretical justifications for the protective role of cultural engagement on depression incidence, to the authors' knowledge, there are currently no studies exploring this relationship using validated depression scales. Consequently, we hypothesised that cultural engagement in older adults is associated with a reduced risk of developing depression over the following decade, and tested this hypothesis using a nationally representative cohort study of older adults in England.

## Method

### Participants

We used data from English Longitudinal Study of Ageing (ELSA): a large, longitudinal cohort study representative of the English population of people aged ≥50 years established in 2002.^23^ The study received ethical approval from the National Research Ethics Service and all participants gave informed consent. We specifically worked with data from wave 2 (2004/2005) across every biennial wave through to wave 7 (2014/2015); a total of six waves and a decade of data.

### Measures

#### Cultural engagement

Our measurement of cultural engagement used self-reports by participants at wave 2 and consisted of three items asking about the frequency of visits to (a) the theatre, concerts or opera, (b) the cinema and (c) an art gallery, exhibition or museum. We combined responses from these three variables to create an overall frequency of receptive cultural engagement, with responses coded as never, less than once a year, once or twice a year, every few months, about once a month or twice a month or more. Because of the small sample size in the two most frequent visits categories, we collapsed these to provide an overall five-point scale.

#### Depression

Depression was measured in two ways. First, we used the Centre for Epidemiologic Studies Depression Scale (CES-D), a widely used self-report measure of depressive symptoms used to identify people at risk of developing depression in the general population.^24^ We specifically used the 8-item version, which has been found to have comparable psychometric properties with the full 20-item scale.^25^ Each item assesses negative affect symptoms or somatic complaints experienced in the past week using a binary reporting scale, with the total number of symptoms summed (0–8). Previous studies using the eight-item CES-D have used a score of three or greater to denote the presence of depression, and this cut-off has been validated against standardised psychiatric interviews with older adults.^25^ To identify whether participants scored above the threshold for depression at any of the waves across the 10 years, we assessed their overall CES-D score at all waves and if a score was three or greater at any wave, they were classed as having experienced a depressive episode.

The second way participants were identified as having experienced depression was if they reported that a doctor had diagnosed them with depression in the 2 years between each wave. This allowed us to identify participants where CES-D scores might have been raised between waves but were recovered by the point of assessment either through intervention (such as antidepressants or counselling) or otherwise.

#### Covariates

We obtained information from wave 2 (baseline) on sociodemographic, health-related and social variables likely to confound associations between exposure and outcome. Sociodemographic covariates included age, gender, ethnicity (coded as White or Black and minority ethnic as ELSA is predominantly White British) and relationship status (in a couple versus without a partner). Socioeconomic position was assessed with net non-pension wealth quintiles, highest educational attainment (no qualifications; educational qualifications at age 16; educational qualifications at age 18; further educational qualifications) and employment status (full time; part time; not in employment).

In relation to participants' health and health behaviours, we assessed whether participants had a chronic/long-standing illness (including cancer, chronic obstructive pulmonary disease, diabetes, angina or a previous stroke), whether they had self-reported problems with eyesight or hearing likely to hinder their participation in cultural activities, and whether they had moderate or severe pain. We also measured self-reported alcohol intake (every couple of months or less; once or twice a month; 1–4 days per week; ≥5 days per week). In addition, we excluded participants registered as blind (*n* = 3) or reporting major difficulties with mobility (unable to walk 91.4 m (100 yards) or sit for 2 h, *n* = 460).

For social variables, we measured social engagement using a composite score of how often participants had contact (whether face to face, over the phone or over email) with friends, children or wider relatives. We assessed whether participants were engaged in any civic activities (including being a member of a political party or environmental group, a tenants or neighbourhood watch group, a church or religious association, a charitable association, an education, arts or music class, a social club, a sports, gym or exercise class or any other society). We also recorded whether participants reported having a hobby or pastime, or reading a daily newspaper.

### Statistical analysis

Incidence rates of depression over the 10 years were computed by frequency of cultural attendance per 100 person-years, calculating the time to onset of depression measured biennially. We then used logistic regression analyses to calculate the odds ratio (OR) and 95% confidence intervals that over the 10 years of follow-up participants experienced a depressive episode. Model 1 adjusted for baseline subclinical CES-D score and demographic covariates: age, gender, marital status, ethnicity, educational attainment, employment status and wealth. Model 2 additionally adjusted for health and health behaviour covariates: eyesight, hearing, chronic health conditions, pain and alcohol consumption. Model 3 additionally adjusted for social covariates: social networks, civic engagement, having a hobby or pastime or reading a daily newspaper. To test for trend, we also modelled cultural engagement as a five-point continuous score, where the odds ratios represent a one-unit change in frequency of engagement.

For all analyses, because of the possibility of left-censoring, whereby participants could enter the study having had depression for many years and have different profiles of cultural engagement, we excluded all participants who had above-threshold depressive symptoms at baseline, who reported visiting a doctor about depression in the 2 years prior to our baseline, who had taken antidepressants or had counselling for depression in the 2 years prior to baseline, or who had an ongoing or recent (past 2 years) diagnosis of any other psychiatric condition (*n* = 335).

We ran a number of sensitivity analyses to test the assumptions of our analyses. We first weighted all data using baseline cross-sectional weights derived from ELSA to ensure the sample was representative of the English population and to account for differential non-response across the following 10 years based on demographic predictors.

The second set of sensitivity analyses explored whether analyses were affected by subclinical symptoms of depression at baseline that might have affected their patterns of cultural engagement or predisposed them to developing depression over the follow-up period by (a) excluding all participants who reported feeling depressed for much of the time over the past week at baseline (even if they had not reported a depression diagnosis or an above-threshold CES-D score); and (b) excluding participants who had a CES-D score of two at baseline indicating possible subclinical symptoms.

The third sensitivity analysis explored the possibility of reverse causality (whereby precursors to the development of depressive symptoms may alter/reduce participation in cultural activities), by conducting a subgroup analysis excluding participants who developed depressive symptoms in the first wave following baseline.

The fourth sensitivity analysis tested whether excluding those with depression at baseline led to a biased sample, so we re-ran analyses including the 335 participants who already had depressive symptoms or a diagnosis of depression at baseline, which allowed us to assess whether cultural participation was associated with the development or continuation of depressive symptoms.

The fifth sensitivity analysis tested the assumption of a threshold of ≥3 on the CES-D scale: as another study has suggested a threshold of ≥4 for identifying people with elevated depression^26^ we re-ran our analyses using this threshold.

Finally, in order to ascertain whether cultural engagement was merely a proxy for having a more open personality type, which itself might have been protective against developing depression, we also controlled for personality using the Midlife Development Inventory personality scale.^27^ Sensitivity analyses are shown in supplementary Tables 1–6 available at https://doi.org/10.1192/bjp.2018.267. All analyses were carried out using Stata SE Version 14.1.

## Results

### Description of the participants

Our sample included a total of 2148 participants. The frequency distribution for the demographic characteristics of the participants is presented in Table 1, along with descriptive data of frequency of cultural engagement. Participants had a mean age of 62.9 years (range 52–89). In total, 74.8% participants reported going to a gallery or museum at least once a year.

**Table 1:** Participant demographics

Covariates all measured at wave 2 (*n* = 2148)	Values
Gender: women, *n* (%)	1108 (51.6)
Age, years: mean (s.d.)	62.9 (7.5)
Ethnicity, Black and minority ethnic: *n* (%)	19 (0.9)
Relationship status, in relationship: *n* (%)	1669 (77.7)
Education, *n* (%)	
NVQ1/CSE or no qualification	547 (25.5)
NVQ2/GCE O level	470 (21.9)
NVQ3 A level/higher education	710 (33.1)
Degree	421 (19.6)
Employment status, *n* (%)	
Not working/retired	1119 (52.1)
Part time	428 (19.9)
Full time ≥35 h/week	601 (28.0)
Health-related issues	
Eyesight problems, *n* (%)	110 (5.1)
Hearing problems, *n* (%)	297 (13.8)
Chronic pain, *n* (%)	46 (2.1)
Chronic illness, *n* (%)	497 (23.1)
Alcohol consumption, *n* (%)	
≥5 days per week	609 (28.4)
1–4 days per week	914 (42.6)
Once or twice a month	257 (12.0)
Every couple of months or less	368 (17.1)
Social isolation score (0–9), mean (s.d.)	4.8 (1.8)
Engaged in civic activities, *n* (%)	399 (18.6)
Have a hobby, *n* (%)	1768 (82.3)
Read a daily newspaper, *n* (%)	1448 (67.4)
Frequency of cultural engagement	
Never	216 (10.1)
Less than once a year	324 (15.1)
Once or twice a year	584 (27.2)
Every few months	635 (29.6)
Once a month or more	389 (18.1)
Number of participants with depression across the 10 years, *n* (%)	616 (28.7)

### Rate of depression by cultural attendance

At baseline, all participants were below the threshold for depression on CES-D, but over the next 10 years, 616 participants (28.7%) recorded a CES-D score above the threshold for depression or reported having been diagnosed since the last wave on at least one occasion. The overall incidence rate was 3.31 (95% CI 3.06–3.58) per 100 person-years. There was an above-average incidence rate for those who never engaged with culture or engaged only infrequently (up to once or twice a year) (Table 2). However, more frequent attendance (every few months or more) was associated with a below-average incidence rate.

**Table 2:** Depression incidence rates per 100 person-years and 95% confidence intervals by frequency of cultural engagement

	New cases of depression in participants over the 10 years, *n*	Rate per 100 person-years (95% CI)
Never	89	5.17 (4.20–6.37)
Less than once a year	104	3.80 (3.14–4.61)
Once or twice a year	174	3.47 (2.99–4.03)
Every few months	167	2.99 (2.57–3.48)
Once a month or more	82	2.31 (1.86–2.87)

### Logistic regression analysis

Engaging with culture every few months or more was associated with a reduced risk of developing depression across the 10 years, independent of demographic factors (model 1), additional health-related factors and behaviours (model 2), and additional social and civic engagement (model 3). There was evidence of a dose–response relationship with more frequent attendance associated with a lower risk. For fully adjusted models, this equated to a 32% lower risk of developing depression for people who attended every few months (OR = 0.68, 95% CI 0.47–0.99, *P* = 0.046) and a 48% lower risk for people who attended once a month or more (OR = 0.52, 95% CI 0.34–0.80, *P* = 0.003) (Table 3).

**Table 3:** Associations between cultural engagement and the risk of developing depression over the following 10 years (*n* = 2148)^a^

	Model 1			Model 2			Model 3		
	OR	95% CI	*P*	OR	95% CI	*P*	OR	95% CI	*P*
Never	Reference			Reference			Reference		
Less than once a year	0.77	0.53–1.13	0.19	0.80	0.54–1.18	0.26	0.80	0.54–1.19	0.27
Once or twice a year	0.71	0.50–1.01	0.060	0.75	0.52–1.07	0.10	0.74	0.51–1.06	0.10
Every few months	**0.65**	**0.45–0.93**	**0.018**	**0.69**	**0.48–1.00**	**0.048**	**0.68**	**0.47–0.99**	**0.046**
Once a month or more	**0.49**	**0.32–0.73**	**0.001**	**0.52**	**0.34–0.79**	**0.002**	**0.52**	**0.34–0.80**	**0.003**

Results in bold are significant.

a. Model 1 adjusted for baseline depressive symptoms, age, gender, marital status, ethnicity, educational attainment, employment status and wealth. Model 2 additionally adjusted for eyesight, hearing, chronic health conditions, pain and alcohol consumption. Model 3 additionally adjusted for social networks, civic engagement, having a hobby or pastime, or reading a daily newspaper.

Tests for trend were significant (OR = 0.87, 95% CI 0.79–0.95, *P* = 0.002). Less frequent attendance (just once or twice a year) appeared to be associated with a reduced risk of depression when adjusting just for sociodemographic factors, but results were attenuated when considering other health and social covariates.

### Sensitivity analyses

Our sensitivity analysis found very similar results when weighting to account for missing data (using fully adjusted models: for every few months, OR = 0.71, 95% CI 0.48–1.05; for once a month or more, OR = 0.53, 95% CI 0.34–0.83) (supplementary Table 1).

When we took into account subclinical symptoms of depression at baseline through exclusions of participants feeling depressed over the previous week or participants with an indication of subclinical CES-D symptoms at baseline, results were materially unaffected (supplementary Table 2).

To test the reverse causal hypothesis of depressive symptoms leading to reduced engagement with culture, we re-ran the regression models excluding the 174 participants who showed above-threshold symptoms of depression in the first wave following baseline. This exclusion had little effect on the estimates (using fully adjusted models: for every few months, OR = 0.67, 95% CI 0.44–1.02; for once a month or more, OR = 0.58, 95% CI 0.36–0.93; supplementary Table 3).

Including participants who already showed depressive symptoms at baseline also did not lead to results being attenuated (using fully adjusted models: for every few months, OR = 0.66, 95% CI 0.47–0.94; for once a month or more, OR = 0.57, 95% CI 0.38–0.84; supplementary Table 4).

Using the alternative cut-off score of ≥4 on CES-D, again results were materially unaffected, although there was a slight loss of power because only 419 participants with depression were detected over the 10 years compared with 616 using the lower threshold (using fully adjusted models: for every few months, OR = 0.67, 95% CI 0.44–1.01; for once a month or more, OR = 0.58, 95% CI 0.37–0.94; supplementary Table 5).

Finally, results were maintained even when adjusting for open personality type (cultural engagement once a month or more, OR = 0.53, 95% CI 0.34–0.82; supplementary Table 6).

## Discussion

### Main findings

The main finding of this study was a dose–response relationship between frequency of cultural engagement and the risk of developing depression over a 10-year period among adults aged ≥50 who were free from depression at baseline. Notably, this finding was independent of sociodemographic factors, health and behavioural factors and other forms of social and civic engagement including other hobbies, social interactions, community group and civic engagement. It was also independent of open personality type.

### Comparisons with findings from other studies

In relation to prior research, this is the first known longitudinal study to explore cultural engagement in relation to the prevention of depression in older age. One previous cross-sectional study found associations between receptive cultural engagement and both low anxiety and low depression (using the Hospital Anxiety and Depression Scale) as well as good satisfaction with life (using a single self-report item).^28^ However, as this study was cross-sectional, it is unclear whether reverse causality was present.

Other cross-sectional studies have, in contrast, found no association between cultural engagement and feelings of anxiety and depression (using the EuroQol-5D).^29^ Two previous longitudinal studies have explored mental health more broadly. A Swedish occupational cohort found weak associations between receptive cultural engagement in the workplace and emotional exhaustion (using the Maslach Burnout Inventory) but not depression symptoms (using the Hopkins Symptom Checklist).^30^ And a Swiss household study found no associations between receptive cultural engagement and either common somatic symptoms (using a cumulative scale similar to the Patient Health Questionnaire) or prevalence of low mood or general life satisfaction (both using a single self-report item).^31^ These studies are also set in the context of others that have found associations between receptive cultural engagement and well-being and life satisfaction.^32^ However, our study is the first to focus specifically on the prevention of depression in older age (rather than the presence of general mental health symptomatology) using a longitudinal sample and a validated depression scale.

### Strengths and limitations

The strengths of this study are that it used well-validated measures of depression and tested different thresholds, finding consistent results. It also used data from a large nationally representative cohort study with consistent collection of key variables every 2 years and a follow-up of a decade. The rich data-set enabled us to include all identified confounding variables in our statistical models.

The main limitation is that this study is observational rather than interventional. We have presented longitudinal associations that attempt to account for issues such as reverse causality and confounding. But causality cannot be assumed and it is possible that residual variables remain. Consequently, interventional studies are recommended as a way of exploring whether cultural engagement could be recommended as an activity to promote positive mental health in older adults and reduce the incidence rate of depression in older adults; especially those identified as being most at risk. Indeed, there have already been calls for more use to be made of cultural venues such as museums and galleries as sites for health promotion and public health interventions,^30^ and these results suggest there could be benefits for mental health.

A further limitation is that it is possible that subthreshold low mood or depression may have contributed to reduced cultural engagement. However, we ran analyses with and without participants with depression at baseline, as well as further excluding participants who had taken antidepressants or had counselling in the past 2 years, who had even very minor symptoms of depression at baseline, who reported feeling low over the past week even if they did not score above-threshold at baseline and who went on to develop depression within 2 years of baseline. None of these additional analyses affected the significance of our results.

Finally, it is possible that a participant experienced a depressive episode in between waves but did not report it to their doctor and recovered by the next CES-D assessment. However, this is anticipated to be a very low number of participants and given the robustness of our findings in response to a range of sensitivity analyses we do not believe this would have affected the broad findings reported here.

### Summary

In conclusion, we found that engagement with cultural activities (including going to the cinema, museums or galleries or the theatre, concert or opera) appears to be an independent risk-reducing factor for the development of depression in older age. Given our analyses specifically tested the potential contribution of reverse causality but found no change in results, this association may be ascribed to multiple components of cultural engagement including social interaction, mental creativity, cognitive stimulation and gentle physical activity.

## Supplementary material

For supplementary material accompanying this paper visit https://doi.org/10.1192/bjp.2018.267. click here to view supplementary material
