# Supplementary material for: Cultural engagement and incident depression in older adults: evidence from the English Longitudinal Study of Ageing
Source: Br J Psychiatry. 2019 Apr;214(4):225–9. doi: 10.1192/bjp.2018.267 (PMC6429253; doi:10.1192/bjp.2018.267)
Supplement: Supplementary file 1 [file S0007125018002672sup001.docx]

# Supplementary Results

**Supplementary Table 1 Results from logistic regression analyses examining the association between cultural engagement and the risk of developing depression at any point over the following 10 years: weighted to account for missing data.**

|  | Model 1 | | | Model 2 | | | Model 3 | | |
| --- | --- | --- | --- | --- | --- | --- | --- | --- | --- |
|  | OR | 95% CI | p | OR | 95% CI | p | OR | 95% CI | p |
| Never | REF | | | REF | | | REF | | |
| ≤Once a year | 0.78 | 0.53-1.16 | .22 | 0.81 | 0.54-1.20 | .29 | 0.81 | 0.54-1.21 | .30 |
| Once or twice a year | **0.70** | **0.49-1.00** | **.048** | 0.73 | 0.50-1.05 | .090 | 0.72 | 0.49-1.05 | .087 |
| Every few months | **0.68** | **0.47-0.98** | **.036** | 0.72 | 0.49-1.05 | .088 | 0.71 | 0.48-1.05 | .087 |
| ≥Once a month | **0.49** | **0.32-0.75** | **.001** | **0.52** | **0.34-0.81** | **.004** | **0.53** | **0.34-0.83** | **.005** |
| N=2,148. Model 1 adjusted for baseline depressive symptoms, age, sex, marital status, ethnicity, educational attainment, employment status and wealth. Model 2 additionally adjusted for eyesight, hearing, chronic health conditions, pain and alcohol consumption. Model 3 additionally adjusted for social networks, civic engagement, having a hobby or pastime or reading a daily newspaper. All weighted using inverse probability weighting. | | | | | | | | | |

**Supplementary Table 2 Results from logistic regression analyses examining the association between cultural engagement and the risk of developing depression at any point over the following 10 years considering symptoms of sub-clinical depression at baseline through (A) excluding participants who had felt depressed in the previous week, or (B) excluding participants with more than 1 sub-clinical symptom at baseline.**

|  | Model 1 | | | Model 2 | | | Model 3 | | |
| --- | --- | --- | --- | --- | --- | --- | --- | --- | --- |
| **(i): N=2,107** | OR | 95% CI | p | OR | 95% CI | p | OR | 95% CI | p |
| Never | REF | | | REF | | | REF | | |
| ≤Once a year | 0.78 | 0.53-1.15 | .21 | 0.81 | 0.55-1.20 | .29 | 0.81 | 0.55-1.20 | .30 |
| Once or twice a year | **0.72** | **0.50-1.02** | **.065** | 0.75 | 0.52-1.08 | .12 | 0.74 | 0.51-1.07 | .11 |
| Every few months | **0.64** | **0.44-0.92** | **.017** | **0.69** | **0.48-1.00** | **.049** | **0.67** | **0.46-0.99** | **.042** |
| ≥Once a month | **0.48** | **0.31-0.72** | **<.001** | **0.51** | **0.33-0.77** | **.002** | **0.51** | **0.33-0.78** | **.002** |
|  | Model 1 | | | Model 2 | | | Model 3 | | |
| **(ii): N=1,914** | OR | 95% CI | p | OR | 95% CI | p | OR | 95% CI | p |
| Never | REF | REF | REF |  |  |  |  |  |  |
| ≤Once a year | 0.71 | 0.47-1.07 | .10 | 0.74 | 0.49-1.12 | .15 | 0.74 | 0.49-1.12 | .15 |
| Once or twice a year | **0.66** | **0.45-0.96** | **.028** | 0.69 | 0.47-1.00 | .052 | 0.68 | 0.46-1.00 | .050 |
| Every few months | **0.60** | **0.41-0.88** | **.009** | **0.64** | **0.43-0.95** | **.026** | **0.64** | **0.42-0.95** | **.028** |
| ≥Once a month | **0.49** | **0.31-0.75** | **.001** | **0.52** | **0.33-0.81** | **.004** | **0.52** | **0.33-0.82** | **.005** |
| Model 1 adjusted for baseline depressive symptoms, age, sex, marital status, ethnicity, educational attainment, employment status and wealth. Model 2 additionally adjusted for eyesight, hearing, chronic health conditions, pain and alcohol consumption. Model 3 additionally adjusted for social networks, civic engagement, having a hobby or pastime or reading a daily newspaper. | | | | | | | | | |
|  | | | | | | | | | |

**Supplementary Table 3 Results from logistic regression analyses examining the association between cultural engagement and the risk of developing depression at any point over the following 10 years: excluding participants who developed depression in wave 3 (the first wave following baseline).**

|  | Model 1 | | | Model 2 | | | Model 3 | | |
| --- | --- | --- | --- | --- | --- | --- | --- | --- | --- |
|  | OR | 95% CI | p | OR | 95% CI | p | OR | 95% CI | p |
| Never | REF | | | REF | | | REF | | |
| ≤Once a year | 0.79 | 0.51-1.21 | .28 | 0.81 | 0.53-1.25 | .34 | 0.82 | 0.53-1.27 | .38 |
| Once or twice a year | 0.73 | 0.49-1.09 | .12 | 0.76 | 0.51-1.13 | .18 | 0.75 | 0.50-1.12 | .16 |
| Every few months | **0.65** | **0.43-0.98** | **.039** | 0.68 | 0.45-1.03 | .071 | 0.67 | 0.44-1.02 | .064 |
| ≥Once a month | **0.55** | **0.35-0.87** | **.011** | **0.58** | **0.37-0.92** | **.020** | **0.58** | **0.36-0.93** | **.024** |
| N=1,974. Model 1 adjusted for baseline depressive symptoms, age, sex, marital status, ethnicity, educational attainment, employment status and wealth. Model 2 additionally adjusted for eyesight, hearing, chronic health conditions, pain and alcohol consumption. Model 3 additionally adjusted for social networks, civic engagement, having a hobby or pastime or reading a daily newspaper. | | | | | | | | | |

**Supplementary Table 4 Results from logistic regression analyses examining the association between cultural engagement and the risk of developing depression at any point over the following 10 years: including participants who showed symptoms of depression at baseline.**

|  | Model 1 | | | Model 2 | | | Model 3 | | |
| --- | --- | --- | --- | --- | --- | --- | --- | --- | --- |
|  | OR | 95% CI | p | OR | 95% CI | p | OR | 95% CI | p |
| Never | REF | | | REF | | | REF | | |
| ≤Once a year | **0.82** | **0.58-1.17** | **.28** | 0.84 | 0.59-1.20 | .34 | 0.84 | 0.59-1.21 | .35 |
| Once or twice a year | **0.70** | **0.50-0.97** | **.035** | 0.73 | 0.52-1.02 | .062 | 0.72 | 0.52-1.01 | .060 |
| Every few months | **0.63** | **0.45-0.88** | **.007** | **0.67** | **0.47-0.94** | **.019** | **0.66** | **0.47-0.94** | **.021** |
| ≥Once a month | **0.53** | **0.36-0.77** | **.001** | **0.55** | **0.38-0.81** | **.003** | **0.57** | **0.38-0.84** | **.005** |
| N=2,468. Model 1 adjusted for baseline depressive symptoms, age, sex, marital status, ethnicity, educational attainment, employment status and wealth. Model 2 additionally adjusted for eyesight, hearing, chronic health conditions, pain and alcohol consumption. Model 3 additionally adjusted for social networks, civic engagement, having a hobby or pastime or reading a daily newspaper. | | | | | | | | | |

**Supplementary Table 5 Results from logistic regression analyses examining the association between cultural engagement and the risk of developing depression at any point over the following 10 years: using a cut off of CES-D≥4.**

|  | Model 1 | | | Model 2 | | | Model 3 | | |
| --- | --- | --- | --- | --- | --- | --- | --- | --- | --- |
|  | OR | 95% CI | p | OR | 95% CI | p | OR | 95% CI | p |
| Never | REF | | | REF | | | REF | | |
| ≤Once a year | 0.70 | 0.46-1.07 | .096 | 0.72 | 0.47-1.10 | .13 | 0.72 | 0.47-1.11 | .14 |
| Once or twice a year | **0.67** | **0.45-0.98** | **.041** | 0.70 | 0.47-1.04 | .078 | 0.69 | 0.46-1.04 | .075 |
| Every few months | **0.63** | **0.42-0.94** | **.023** | 0.68 | 0.45-1.01 | .058 | 0.67 | 0.44-1.01 | .058 |
| ≥Once a month | **0.54** | **0.35-0.86** | **.008** | **0.58** | **0.37-0.92** | **.02** | **0.58** | **0.37-0.94** | **.025** |
| N=2,148. Model 1 adjusted for baseline depressive symptoms, age, sex, marital status, ethnicity, educational attainment, employment status and wealth. Model 2 additionally adjusted for eyesight, hearing, chronic health conditions, pain and alcohol consumption. Model 3 additionally adjusted for social networks, civic engagement, having a hobby or pastime or reading a daily newspaper. | | | | | | | | | |

**Supplementary Table 6 Results from logistic regression analyses examining the association between cultural engagement and the risk of developing depression at any point over the following 10 years: additionally adjusting for personality**

|  | Model 1 | | | Model 2 | | | Model 3 | | |
| --- | --- | --- | --- | --- | --- | --- | --- | --- | --- |
|  | OR | 95% CI | p | OR | 95% CI | p | OR | 95% CI | p |
| Never | REF | | | REF | | | REF | | |
| ≤Once a year | 0.72 | 0.48-1.07 | .10 | 0.75 | 0.50-1.12 | .16 | 0.74 | 0.49-1.12 | .15 |
| Once or twice a year | **0.71** | **0.49-1.03** | **.075** | 0.75 | 0.51-1.09 | .13 | 0.73 | 0.50-1.07 | .10 |
| Every few months | **0.67** | **0.46-0.98** | **.037** | 0.71 | 0.48-1.04 | .079 | 0.69 | 0.47-1.02 | .064 |
| ≥Once a month | **0.50** | **0.33-0.77** | **.002** | **0.53** | **0.34-0.81** | **.004** | **0.53** | **0.34-0.82** | **.005** |
| N=2,148. Model 1 adjusted for baseline depressive symptoms, age, sex, marital status, ethnicity, educational attainment, employment status and wealth. Model 2 additionally adjusted for eyesight, hearing, chronic health conditions, pain and alcohol consumption. Model 3 additionally adjusted for social networks, civic engagement, having a hobby or pastime or reading a daily newspaper. All three models were adjusted for open personality type. | | | | | | | | | |
